# MRI-detected brain lesions in AF patients without further stroke risk factors undergoing ablation - a retrospective analysis of prospective studies

**DOI:** 10.1186/s12872-019-1035-1

**Published:** 2019-03-12

**Authors:** Juliane Herm, Johannes Schurig, Martin R. Martinek, Reinhard Höltgen, Alexander Schirdewan, Paulus Kirchhof, Marcus Wieczorek, Helmut Pürerfellner, Peter U. Heuschmann, Jochen B. Fiebach, Karl Georg Haeusler

**Affiliations:** 1 Department of Neurology, Charité - Universitätsmedizin Berlin, Berlin, Germany; 2 Center for Stroke Research Berlin, Charité - Universitätsmedizin Berlin, Berlin, Germany; 3 Department of Cardiology, Ordensklinikum Linz Elisabethinen, Linz, Austria; 4 Department of Cardiology and Electrophysiology, St. Agnes-Hospital Bocholt, Bocholt, Germany; 5 Department of Cardiology, Sana Clinic Lichtenberg, Berlin, Germany; 6 Institute of Cardiovascular Sciences, University of Birmingham, Birmingham, UK; 7 Witten/Herdecke University, School of Medicine, Witten, Germany; 8 Institute of Clinical Epidemiology and Biometry, University of Würzburg, Würzburg, Germany; 9 Clinical Trial Center Würzburg, University Hospital Würzburg, Würzburg, Germany; 10 Comprehensive Heart Failure Center, University of Würzburg, Würzburg, Germany; 11 Department of Neurology, Universitätsklinikum Würzburg, Josef-Schneider-Str. 11 97080, Würzburg, Germany

**Keywords:** Clinically silent stroke - atrial fibrillation - magnetic resonance imaging - cerebral microbleeds

## Abstract

**Background:**

Atrial fibrillation (AF) without other stroke risk factors is assumed to have a low annual stroke risk comparable to patients without AF. Therefore, current clinical guidelines do not recommend oral anticoagulation for stroke prevention of AF in patients without stroke risk factors. We analyzed brain magnetic resonance imaging (MRI) imaging to estimate the rate of clinically inapparent (“silent”) ischemic brain lesions in these patients.

**Methods:**

We pooled individual patient-level data from three prospective studies comprising stroke-free patients with symptomatic AF. All study patients underwent brain MRI within 24–48 h before planned left atrial catheter ablation. MRIs were analyzed by a neuroradiologist blinded to clinical data.

**Results:**

In total, 175 patients (median age 60 (IQR 54–67) years, 32% female, median CHA_2_DS_2_-VASc = 1 (IQR 0–2), 33% persistent AF) were included. In AF patients without or with at least one stroke risk factor, at least one silent ischemic brain lesion was observed in 4 (8%) out of 48 and 10 (8%) out of 127 patients, respectively (*p* > 0.99). Presence of silent ischemic brain lesions was related to age (*p* = 0.03) but not to AF pattern (*p* = 0.77). At least one cerebral microbleed was detected in 5 (13%) out of 30 AF patients without stroke risk factors and 25 (25%) out of 108 AF patients with stroke risk factors (*p* = 0.2). Presence of cerebral microbleeds was related to male sex (*p* = 0.04) or peripheral artery occlusive disease (*p* = 0.03).

**Conclusion:**

In patients with symptomatic AF scheduled for ablation, brain MRI detected silent ischemic brain lesions in approximately one in 12 patients, and microbleeds in one in 5 patients. The prevalence of silent ischemic brain lesions did not differ in AF patients with or without further stroke risk factors.

**Electronic supplementary material:**

The online version of this article (10.1186/s12872-019-1035-1) contains supplementary material, which is available to authorized users.

## Background

Atrial fibrillation (AF) is the most common cardiac arrhythmia worldwide causing about 15–20% of all ischemic strokes. Individual stroke risk is determined by the presence of cardiovascular risk factors such as arterial hypertension, old age and prior stroke [1]. About 3.5% of all AF patients do not have coexisting cardiovascular risk factors (“lone” AF) [2]. Based on a reported thromboembolic event rate of 0.4–0.8% per year, AF without stroke risk factors is considered to be a “benign” disease [3, 4] and oral anticoagulation is not recommended in these patients for secondary stroke prevention [1]. According to an observational study, the prevalence of fibrotic changes in the left atrium – which is linked to stroke risk [5] – was reported to be similar in AF patients with and without coexisting cardiovascular risk factors [6] but reported findings are inconsistent [7].

Brain magnetic resonance imaging (MRI) may help to better quantify the stroke risk of AF patients without stroke risk factors by assessing clinically “silent” ischemic brain lesions, known to be related to clinical evident stroke [8, 9] and dementia [10, 11]. In addition, MRI quantifies the microangiopathic lesion load - so called “white matter hyperintensities” - an established marker of small-vessel disease and linked to AF-related cerebral hypoperfusion, endothelial dysfunction or embolism to the brain [12, 13]. Moreover, brain MRI is capable to detect small hypointense T2*-lesions (so called “cerebral microbleeds”), a known risk factor for microangiopathic disease and intracerebral hemorrhage [14, 15].

In order to analyze whether silent ischemic brain lesions are found more frequently in AF patients with at least one stroke risk factor compared to those without stroke risk factors, we retrospectively analyzed individual patient data assessed in three prospective monocenter studies focusing on MRI-detected brain lesions after left atrial catheter ablation for symptomatic AF in Germany or Austria [16, 17]. Furthermore, we compared the frequency of cerebral microbleeds or white matter hyperintensities (WMH) in AF patients with or without stroke risk factors.

## Methods

### Study design and study population

According to the respective study protocols, all three studies recruited consecutive patients presenting for pulmonary vein isolation in symptomatic AF. Patients were eligible for the present analysis if they had symptomatic non-permanent AF and no history of prior stroke or transient ischemic attack (TIA). Focusing on silent brain lesions after ablation, all patients underwent “baseline” brain MRI within 24–48 h prior to scheduled ablation. All patients underwent echocardiography according to study protocols in order to rule out a thrombus before ablation. Left atrial dimensions were determined in 165 (94.3%) patients using the parasternal view in 81% and the apical 4-chamber view in 19%. A dilated left atrium was therefore defined as ≥42 mm in diameter (parasternal view) or ≥ 58 mm (long axis, apical view) [18, 19]. In all three studies, demographic data including stroke risk factors were assessed.

The present data pooling has been approved by the Ethics Committee of the Charité –Universitätsmedizin Berlin, Germany (EA4/087/08). Furthermore, each individual study was approved by the local Ethics Committee before (Ethics Committee Witten/Herdecke, Germany (114/2016); Ethics Committee of the Elisabethinen University Teaching Hospital Linz, Austria (K-103-16)). Individual patient data were taken from the MACPAF study (*n* = 35; [16]), the Austrian study (*n* = 116; [17]) and a study in Bocholt, Germany (*n* = 24).

### Brain imaging

Using a 3 Tesla MR scanner (Siemens, Erlangen, Germany) in Berlin; Germany [16] and a 1.5 Tesla MR scanner (Siemens, Erlangen, Germany) in Linz, Austria and Bocholt, Germany, the following MRI sequences were assessed in 175 study patients: diffusion-weighted magnetic resonance imaging (DWI; *n* = 175) to identify acute ischemic brain lesions; T2*-weighted imaging (*n* = 138) to diagnose microbleeds and exclude brain hemorrhage, and Fluid-attenuated inverse recovery (FLAIR, *n* = 58) or conventional T2 weighted images (*n* = 117) to assess the microangiopathic load. Evaluation of all MRI images was done by a board certified neuroradiologist (JBF), blinded to all clinical data White matter hyperintensities (WMH) were graded according to the Fazekas score [20]. A cerebral microbleed was defined as a small (≤10 mm), hypointense lesion in T2*-weighted imaging. The nomenclature endorsed by STRIVE was used as applicable [21]. To allow a comparison of reported brain MRI data to patients without AF, a subgroup of volunteers enrolled to the prospective single-center “Berlin Beat of Running study” [22] was analyzed. An identical MRI protocol was used in the “Berlin Beat of Running study” and in the MACPAF study.

### Statistical analysis

Absolute and relative frequencies were reported for categorical variables. As a result of comparably small sample sizes, normal distribution of continuous variables could not be assumed. Therefore, medians and quartiles were calculated for all variables. The Fisher exact test was used to compare proportions of dichotomous outcomes between independent groups. The Mann-Whitney test was used to compare proportions of nominal variables between independent groups. Impact factors regarding the burden of cerebral microbleeds were reported using a Poisson regression model. A *p* < 0.05 indicates significant associations.

## Results

Baseline data of all 175 study patients are shown in Table 1. Median age at enrolment was 60 (IQR 54–67) years, 32% of all patients were female. Median CHA_2_DS_2_-VASc-score was 1.0 (IQR 0.0–2.0, range 0–5). Overall, 48 (27%) patients presented without further stroke risk factors (defined as a CHA_2_DS_2_-VASc-score = 0 in men (*n* = 38) or = 1 in women (*n* = 10)). Comparing patients across studies, patients recruited in Bocholt, Germany had more frequently congestive heart failure compared to MACPAF patients enrolled in Berlin, Germany (21% vs. 2%, *p* = 0.04) or patients enrolled in Linz, Austria (21% vs. 6%, *p* = 0.02), respectively. Moreover, all MACPAF patients had paroxysmal AF (defined inclusion criterion), whereas 61% of all patients enrolled in Linz, Austria and 50% of all patients enrolled in Bocholt presented with paroxysmal AF (*p* < 0.001). Age, sex and further cardiovascular risk factors did not differ across studies (see Additional file 1).

**Table 1:** Baseline characteristics of 175 study patients with AF but without known ischemic stroke

	All study	AF without stroke risk factors		
	patients *n* = 175	No (*n* = 48)	Yes (*n* = 127)	p*
Age; years, median (IQR)	60.0 (54.0–67.0)	56.5 (49.3–60.8)	64.0 (55.0–67-0)	–
Female sex; n (%)	56 (32.0)	10 (20.8)	46 (36.2)	–
Congestive heart failure; n (%)	14 (8.0)	–	14 (11.0)	–
Arterial hypertension; n (%)	100 (57.1)	–	100 (78.7)	–
Diabetes mellitus; n (%)	17 (9.7)	–	17 (13.4)	–
Peripheral artery occlusive disease; n (%)	4 (2.3)	–	4 (3.1)	–
Coronary artery disease; n (%)	33 (18.9)	–	33 (26.0)	–
Cardiomyopathy; n (%)	9 (6.0)	2 (4.5)	7 (6.5)	> 0.999
Hyperlipoproteinemia; n (%)	87 (49.7)	18 (37.5)	69 (54.3)	0.062
Dilated left atrium; n (%)	75 (45.5)	14 (31.8)	61 (50.4	0.036
Paroxysmal AF	118 (67.4)	36 (75.0)	82 (64.6)	0.210

### MRI-detected ischemic brain lesions

**Fig. 1:**
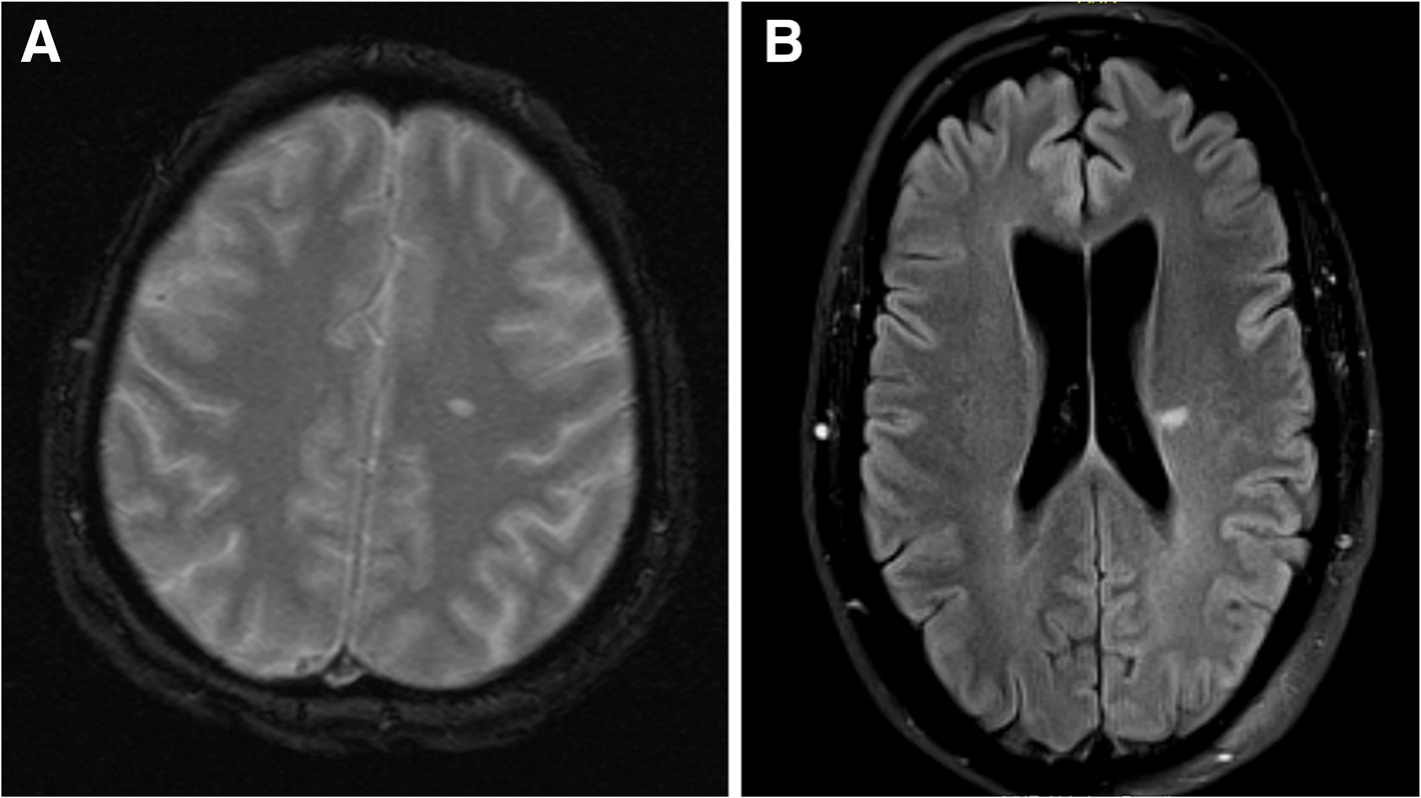
**a** Conventional T2-weighted imaging showing a single ischemic brain lesion in a female AF patient aged 63 years without stroke risk factors and (**b**) Fluid-attenuated inverse recovery (FLAIR) showing a single ischemic brain lesion in a male AF patient aged 50 years without stroke risk factors

Brain MRI before planned ablation detected acute (*n* = 2) or chronic (*n* = 12) silent ischemic brain lesions in 14 (8%) patients without a history of stroke (Fig. 1). Silent ischemic brain lesions were classified as territorial stroke (*n* = 7) or small subcortical singular lesions without surrounding white matter disease (*n* = 6) or with surrounding white matter disease (“lacunes of presumed vascular origin”; n = 1). In AF patients, silent ischemic lesions were observed in 4 (8%) out of 48 AF patients without stroke risk factors compared to 10 out of 127 (8%) AF patients with at least one stroke risk factor (*p* > 0.99). (Table 2). The median CHA_2_DS_2_-VASc-score did not differ in patients with and without MRI-detected silent ischemic brain lesions (*p* = 0.43). In more detail, 3 (5%) out of 58 patients with a CHA_2_DS_2_-VASc score of 1 (excluding female sex), 3 (8%) out of 40 patients with a CHA_2_DS_2_-VASc score of 2 (excluding female sex) and 4 (14%) of 29 patients with a CHA_2_DS_2_-VASc score of ≥3 (excluding female sex) had MRI-detected silent ischemic brain lesions. Old age was associated with the presence of ischemic lesions (*p* = 0.03), while individual stroke risk factor(s) such as diabetes and arterial hypertension as well as sex, AF type or left atrial diameter were not (Table 2).

**Table 2:** Cardiovascular risk profile of study patients with and without MRI-detected silent ischemic lesions

		Silent ischemic brain lesions		
		No (*n* = 161)	Yes (*n* = 14)	*p*-value*
≥1 stroke risk factor**	No	44 (27.3)	4 (28.6)	> 0.999
	Yes	137 (72.7)	10 (71.4)	
Age; median (IQR)		60 (54–66)	67 (61–69)	0.026
Female sex; n (%)		50 (31.1)	6 (42.9)	0.318
Congestive heart failure; n (%)		11 (6.8)	1 (7.1)	> 0.999
Arterial hypertension; n (%)		94 (58.4)	6 (42.9)	0.275
Diabetes mellitus; n (%)		15 (9.3)	2 (14.5)	0.630
Vascular disease; n (%)		34 (21.1)	1 (7.1)	0.307
Hyperlipoproteinemia; n (%)		77 (47.8)	10 (71.4)	0.103
Paroxysmal AF; n (%)		109 (67.7)	9 (64.3)	0.773
Dilated left atrium; n (%)		70 (45.8)	5 (41.7)	> 0.999
Left ventricular ejection fraction < 50%; n (%)		11 (7.0)	0 (0)	> 0.999

* *p*-value calculated by chi^2^-test or Mann-Whitney U test, as appropriate. ** According to CHA_2_DS_2_-VASc-score (“No” equals 0 in men and 1 in women, “Yes” equals ≥1 in men and ≥ 2 in women)

To compare our cohort of 48 AF patients without stroke risk factors to a healthy population without AF, we identified an age- and sex-matched subgroup of the “Berlin Beat of Running study”, enrolling (non-professional) healthy endurance athletes aged 35 to 60 years before running a marathon. In 37 volunteers (median 54 years, range 39–60 year; 22% female), 3 T MRI identified no silent ischemic lesions (0% vs. 8% in AF patients without stroke risk factors (median 57 years, range 27–64 years; 21% female); *p* = 0.129)).

### MRI-detected white matter hyperintensities (WMH)

WMH were found in 81 (46%) of all 175 study patients and in 19 (40%) of 48 AF patients without stroke risk factors. The Fazekas score was equally distributed in AF patients with or without stroke risk factor(s) (median 0.0 (IQR 0.0–1.0, range 0–2) vs. median 0.0 (IQR 0.0–1.0, range 0–2; *p* = 0.26). The Fazekas score was higher in patients with MRI-detected cerebral microbleeds compared to patients without cerebral microbleeds (median 1.0 (IQR 0.0–1.0) vs. 0.0 (IQR 0.0–1.0); *p* = 0.01) as well as in patients with MRI-detected ischemic lesions compared to those without (median 1.0 (IQR 0.8–1.0) vs. 0.0 (IQR 0.0–1.0); *p* = 0.02). Old age (*p* < 0.01) was associated with MRI-detected WMH, while other individual stroke risk factors such as arterial hypertension, diabetes as well as sex were not (Table 3).

**Table 3:** Cardiovascular risk profile of patients with or without white matter hyperintensities (WMH) according to brain MRI

		WMH*		
		No (*n* = 94)	Yes (*n* = 81)	*p*-value**
≥1 stroke risk factor***	No	29 (30.9)	19 (23.5)	0.310
	Yes	65 (69.1)	62 (76.5)	
Age; median (IQR)		58 (52–64)	64 (57–69)	< 0.001
Female sex; n (%)		29 (30.9)	27 (33.3)	0.747
Congestive heart failure; n (%)		6 (6.4)	6 (7.4)	> 0.999
Arterial hypertension; n (%)		53 (56.4)	47 (58.0)	0.879
Diabetes mellitus; n (%)		8 (8.5)	9 (11.1)	0.615
Peripheral artery occlusive disease; n (%)		2 (2.1)	2 (2.5)	> 0.999
Coronary artery occlusive disease; n (%)		15 (16.0)	18 (22.2)	0.335
Hyperlipoproteinemia; n (%)		43 (45.7)	44 (54.3)	0.290
Paroxysmal AF; n (%)		57 (60.6)	61 (75.3)	0.052
Dilated left atrium according to echocardiography; n (%)		39 (43.8)	36 (47.4)	0.754
Left ventricular ejection fraction < 50%; n (%)		5 (5.4)	6 (7.6)	0.756

* WMH were determined according to Fazekas score. ** p-value calculated by chi ^*2*^ -test or Mann-Whitney U test, as appropriate. *** According to CHA_*2*_ *DS*_*2*_ *-VASc-score (“No” equals 0 in men and 1 in women, “Yes” equals ≥ 1 in men and ≥ 2 in women)*

### MRI-detected cerebral microbleeds

**Fig. 2:**
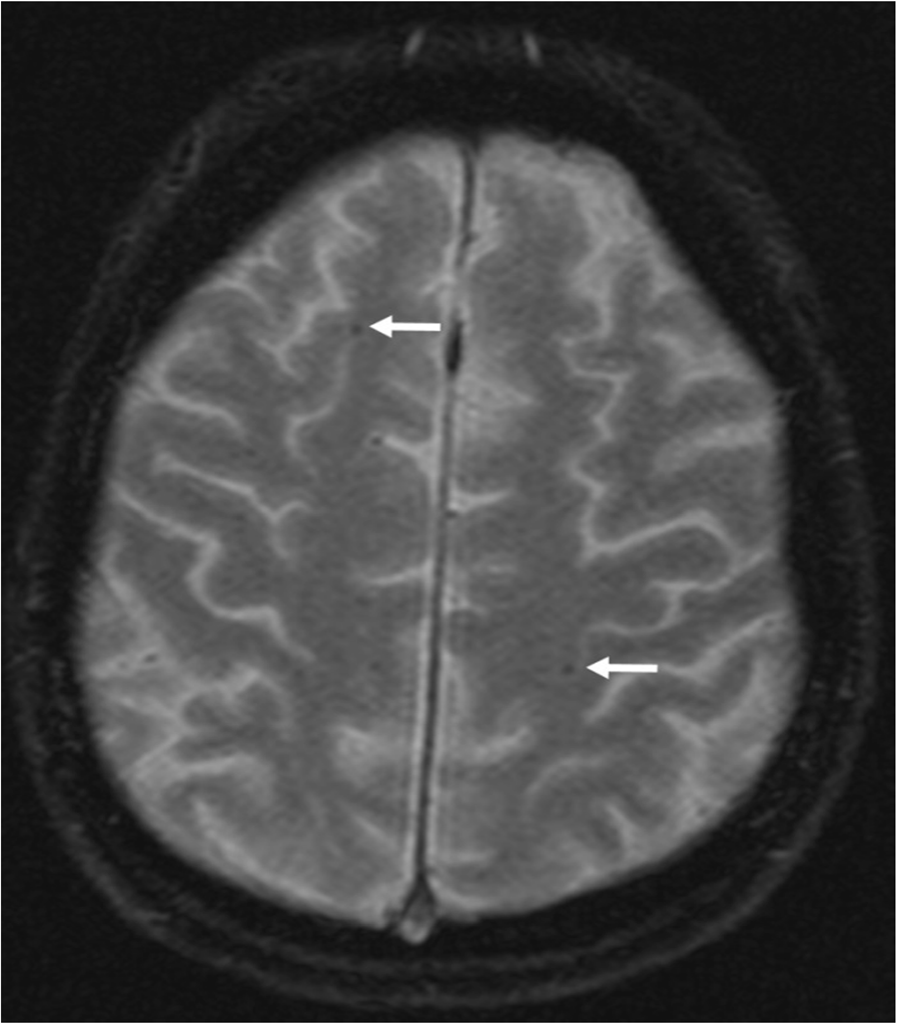
T2*-weighted imaging depicting multiple cerebral microbleeds (indicated by arrows) in a man aged 71 years with known AF and coronary artery occlusive disease (CHA_2_DS_2_-VASc-score = 2)

T2*-weighted imaging was performed in 138 (79%) out of all 175 study patients. Comparing patients with T2*-weighted imaging to those without, the prevalence of stroke risk factors and the distribution of age and sex did not differ. Compared to those patients without T2*-weighted imaging, patients with T2*-weighted imaging more often had persistent AF (41% vs. 3%, *p* < 0.01). All 138 patients received oral anticoagulation (using phenprocoumon (*n* = 122) or a non-vitamin-K dependent oral anticoagulant (*n* = 16)) for at least six weeks prior to ablation according to the local study protocol [17]. Brain MRI detected at least one cerebral microbleed in 30 (22%) patients, 6 (4%) had more than one cerebral microbleed (median 1.0 (IQR 1.0–1.0, range 1–4)) (Fig. 2**,** Table 4). Location of supratentorial cerebral microbleeds was strictly lobar in 23 (77%) patients, strictly deep in 3 (9%) patients and mixed in 1 (3%) patient (Table 5). In AF patients without stroke risk factors, cerebral microbleeds were observed in 5 (13%) out of 39 compared to 25 (25%) out of 99 in AF patients with stroke risk factors (*p* = 0.17). There was a statistical non-significant trend towards a lower burden of cerebral microbleeds in AF patients without stroke risk factors (median 0.0 (IQR 0.0–0.0) vs. median 0.0 (IQR 0.0–1.0) in AF patients with stroke risk factors (*p* = 0.09)). Male sex (*p* = 0.04) and peripheral artery occlusive disease (*p* = 0.03) were associated with the presence of MRI-detected cerebral microbleeds, while diabetes, arterial hypertension, AF pattern and age were not. Using a Poisson regression model, male sex (OR 4.3 (95%CI 1.4–12.6)) and patients age (OR 1.8 per decade (95%CI 1.1–2.7)) were associated with a higher burden of cerebral microbleeds, while diabetes, arterial hypertension, peripheral artery occlusive disease, coronary artery disease or congestive heart failure were not (data not shown).

**Table 4:** Cardiovascular risk profile of patients with or without cerebral microbleeds (CMB) according to available MRI data (*n* = 138)

		CMB		
		No (*n* = 108)	Yes (*n* = 30)	*p*-value*
≥1 stroke risk factor**	No	34 (31.5)	5 (16.7)	0.168
	Yes	74 (68.5)	25 (83.3)	
• Age; median (IQR)		59 (53–65)	64 (56–67)	0.098
Female sex; n (%)		37 (34.3)	4 (13.3)	0.040
Congestive heart failure; n (%)		9 (8.3)	2 (6.7)	> 0.999
Arterial hypertension; n (%)		60 (55.6)	18 (60.0)	0.684
Diabetes mellitus; n (%)		9 (8.3)	3 (10.0)	0.769
Peripheral artery occlusive disease; n (%)		1 (0.9)	3 (10.0)	0.032
Coronary artery occlusive Disease; n (%)		18 (16.7)	6 (20.0)	0.786
Hyperlipoproteinemia; n (%)		51 (47.2)	17 (56.7)	0.412
Paroxysmal AF, n (%)		70 (58.8)	19 (59.4)	> 0.999
INR > 3.0 prior to ablation, n (%)		16 (14.8)	3 (10.0)	0.765
Intake of phenprocoumon^#^		93 (86.1)	29 (96.6)	0.194
Intake of NOAK^#,^ ***		15 (13.9)	1 (3.3)	

* p-value calculated by chi ^*2*^ -test or Mann-Whitney U test, as appropriate. Multivariate analysis was calculated in a binary logistic regression model using backward selection. ** According to CHA_*2*_ *DS*_*2*_ *-VASc-score (“No” equals 0 in men and 1 in women, “Yes” equals ≥ 1 in men and ≥ 2 in women).* ^*#*^ at the time of study enrollment. *** Non-Vitamin-K dependent oral anticoagulant

**Table 5:** Distribution of MRI-detected CMB in AF patients with and without stroke risk factors and without prior stroke according to medical history (*n* = 30)

	AF without stroke risk factors	
	Yes (*n* = 5)	No (*n* = 25)
Multiple CMB; n (%)	0 (0)	6 (24.0)
Left hemisphere; n (%)	3 (60.0)	11 (44.0)
Both hemispheres; n (%)	0 (0)	4 (16.0)
Strictly lobar; n (%)	5 (100.0)	18 (72.0)
Strictly deep; n (%)	0 (0)	3 (12.0)
Mixed (lobar and deep); n (%)	0 (0)	1 (4.0)
Infratentorial region; n (%)	0 (0)	4 (16.0)

## Discussion

The main finding of this retrospective analysis of patient-level data assessed within three prospective monocenter studies is that the prevalence of MRI-detected silent ischemic brain lesions as well as the burden of cerebral microangiopathy is similar in AF patients with or without stroke risk factors. The burden of cerebral microbleeds was comparably low in AF patients without stroke risk factors, which was not reported before.

According to guideline recommendations, the clinical impact of MRI-detected silent ischemic brain lesions in AF patients is low [1]. However, a MRI-based cohort study including 400 AF patients demonstrated an elevated risk for later ischemic stroke in patients with MRI-detected silent ischemic brain lesions [23]. Therefore, one might argue that MRI-detected silent ischemic brain lesions should have similar implications as a transient ischemic attack (TIA) diagnosed on clinical grounds alone. While published cohort studies and a meta-analysis on brain MRI findings in unselected AF patients reported a prevalence of silent ischemic brain lesions of 28–90% [24–26], a cohort study including 38 AF patients with a CHA_2_DS_2_-VASc-score of 0 reported MRI-detected silent ischemic brain lesions in 3% in these patients [23]. We observed at least one silent ischemic brain lesion in 8% of all 175 patients, similarly distributed in AF patients with (8%) or without at least one stroke risk factor (8%, *p* > 0.99). Of note, silent ischemic brain lesions were either territorial strokes or small subcortical singular lesions, thus indicating predominantly embolic sources as previously discussed in AF patients [25]. Of note, comparing our cohort of 48 AF patients without stroke risk factors to 37 age- and sex-matched individuals without stroke risk factors, a subgroup of the prospective “Berlin Beat of Running study”, 3 T MRI identified no silent ischemic lesions. However, as the “Berlin Beat of Running Study” had enrolled only marathon participants aged 35–60 years, we cannot draw definitive conclusions regarding the prevalence of silent ischemic brain lesions in non-AF patients over 60 years of age.

Focusing on white matter lesions, a low microangiopathic lesion load was present in the entire study cohort, which is in line with the observed cardiovascular risk profile (Table 1). However, at least a single microangiopathic brain lesion was found in in 46% of all 175 AF-patients without prior stroke. Not focusing AF patients without stroke risk factors, a cohort study reported white matter lesions in 68% of 74 AF patients (median Fazekas 1, median CHA_2_DS_2_-VASc-score 2, median age 59 years) without prior stroke or TIA planned for left atrial catheter ablation [27]. As the prevalence of peripheral artery occlusive disease and diabetes was considerably lower in our cohort than in this Polish population, this may account for the lower number of patients with white matter lesions. Comparing AF patients with or without stroke risk factors, the Fazekas score was equally distributed in our cohort (median 0 (IQR 0–1) vs. median 0 (IQR 0–1), *p* = 0.26).

A recent meta-analysis demonstrated a 4-fold increased risk of future intracerebral hemorrhage in AF patients with ischemic stroke and MRI-detected cerebral microbleeds [28]. In AF patients with clinically evident ischemic stroke, at least one cerebral microbleed has been reported in 22–49% of patients [29, 30]. We observed at least one cerebral microbleed in 13% of AF patients without stroke risk factors and in 25% of AF patients with stroke risk factors (*p* = 0.17; Table 4). There was a non-significant trend towards a higher burden of cerebral microbleeds if present in AF patients with stroke risk factors compared to those without (*p* = 0.09). Study patients with a higher burden of cerebral microbleeds were more often male and older compared to study patients without cerebral microbleeds.

Our study has several limitations. First, this is a retrospective analysis of individual patient data acquired in prospective studies with a slightly different MRI protocol- using 1.5 Tesla (*n* = 140) or 3 Tesla (*n* = 35) which may impact on the detection of acute ischemic lesions [31]. Unfortunately, a sensitivity analysis is not applicable due to the limited number of patients undergoing MRI at 3 Tesla. Second, follow-up data are missing, but catheter ablation per se may have a significant impact on further MRI findings [32, 33]. Third, duration of oral anticoagulation prior investigation may have differed in our study patients with or without stroke risk factors and may have an impact on the presence of MRI-detected cerebral microbleeds [34]. Fourth, all study patients had symptomatic AF and were scheduled to undergo left atrial catheter ablation. Therefore, the generalizability of our data is limited to certain extent and further studies in AF patients without stroke risk factors are needed to validate or findings. Finally, the sample size is rather small, although this is obviously the largest published sample up to date.

## Conclusion

Our data indicate that MRI-detected silent ischemic brain lesions are a frequent finding in patients scheduled for ablation due to symptomatic AF. Of note, the prevalence of silent ischemic brain lesions was similar in AF patients with or without further stroke risk factors, respectively. While the MRI-detected burden of microangiopathic lesions was rather low in both cohorts, there was a trend for a lower prevalence of cerebral microbleeds in AF patients without stroke risk factors. Further studies are warranted to validate our findings in AF patients scheduled for ablation or in AF patients per se.

## Additional file

Additional file 1: **Table S1.** Online Supplement: Baseline characteristics of 175 study patients according to study center. Description of data: This table contains the baseline characteristics of 175 study patients according to study center (Berlin, Linz and Bocholt). (DOCX 14 kb)

## Abbreviations

AF: Atrial fibrillation
MRI: magnetic resonance imaging
WMH: white matter hyperintensities
