# Supplementary material for: MRI-detected brain lesions in AF patients without further stroke risk factors undergoing ablation - a retrospective analysis of prospective studies
Source: BMC Cardiovasc Disord. 2019 Mar 12;19:58. doi: 10.1186/s12872-019-1035-1 (PMC6419420; doi:10.1186/s12872-019-1035-1)
Supplement: Supplementary file 1 — Table S1. Online Supplement: Baseline characteristics of 175 study patients according to study center. Description of data: This table contains the baseline characteristics of 175 study patients according to study center (Berlin, Linz and Bocholt). (DOCX 14 kb) [file 12872_2019_1035_MOESM1_ESM.docx]

**Table 1 Online Supplement:** Baseline characteristics of 175 study patients according to study center.

|  | **Berlin**  n=35 | **Bocholt**  (n=24) | **Linz**  (n=116) | **p***  **Berlin vs. Bocholt** | **p***  **Berlin vs. Linz** | **p***  **Bocholt vs. Linz** |
| --- | --- | --- | --- | --- | --- | --- |
| Age; years, median (IQR) | 63 (56-68) | 59 (50-68) | 60 (54-66) | 0.187 | 0.089 | 0.747 |
| Female sex; n (%) | 14 (40.0) | 8 (36.4) | 34 (29.3) | 0.785 | 0.228 | 0.807 |
| Congestive heart failure; n (%) | 1 (2.1) | 5 (20.8) | 6 (5.2) | **0.036** | >0.999 | **0.022** |
| Arterial hypertension; n (%) | 20 (51.7) | 15 (62.5) | 65 (46.0) | 0.790 | 0.849 | 0.653 |
| Diabetes mellitus; n (%) | 5 (14.3) | 4 (16.7) | 8 (6.9) | >0.999 | 0.306 | 0.126 |
| Peripheral artery occlusive disease; n (%) | 0 (0) | 0 (0) | 4 (3.4) | - | 0.573 | >0.999 |
| Coronary artery disease; n (%) | 8 (22.9) | 2 (8.3) | 23 (19.8) | 0.177 | 0.643 | 0.247 |
| Cardiomyopathy; n (%) | 3 (8.6) | 5 (20.8) | 2 (1.7) | 0.251 | 0.087 | **0.002** |
| Paroxysmal AF | 35 (100.0) | 12 (50.0) | 71 (61.2) | **<0.001** | **<0.001** | 0.364 |
